# Author Correction: Ocean fronts and eddies force atmospheric rivers and heavy precipitation in western North America

**DOI:** 10.1038/s41467-026-68408-1

**Published:** 2026-01-30

**Authors:** Xue Liu, Xiaohui Ma, Ping Chang, Yinglai Jia, Dan Fu, Guangzhi Xu, Lixin Wu, R. Saravanan, Christina Marie Patricola-DiRosario

**Affiliations:** 1 International Laboratory for High-Resolution Earth System Prediction, Texas A&M University, College Station, TX USA; 2 Department of Oceanography, Texas A&M University, College Station, TX USA; 3 Key Laboratory of Physical Oceanography and Frontiers Science Center for Deep Ocean Multispheres and Earth System, Ocean University of China, Qingdao, China; 4 Qingdao Pilot National Laboratory for Marine Science and Technology, Qingdao, China; 5 Department of Atmospheric Sciences, Texas A&M University, College Station, TX USA; 6 College of Global Change and Earth System Science, Beijing Normal University, Beijing, China; 7 Department of Geological and Atmospheric Sciences, Iowa State University, Ames, IA USA

Correction to: *Nature Communications* 10.1038/s41467-021-21504-w, published online 24 February 2021

Since the publication of this work, Christina Marie Patricola-DiRosario has changed their name from Christina M. Patricola. This has now been amended in the HTML and PDF versions of the article.

