# Supplementary material for: Ocean fronts and eddies force atmospheric rivers and heavy precipitation in western North America
Source: Nat Commun. 2021 Feb 24;12:1268. doi: 10.1038/s41467-021-21504-w (PMC7904778; doi:10.1038/s41467-021-21504-w)
Supplement: Supplementary file 1 — Supplementary Information [file 41467_2021_21504_MOESM1_ESM.pdf]

Supplementary Information for

Ocean Fronts and Eddies Force Atmospheric Rivers and Heavy Precipitation in Western North America

Xue Liu[1,2,8], Xiaohui Ma[3,4,8*], Ping Chang[1,2,5], Yinglai Jia[3], Dan Fu[1,2], Guangzhi Xu[6], Lixin Wu[3,4], R. Saravanan[1,5], Christina M. Patricola[7]

1. International Laboratory for High-Resolution Earth System Prediction, Texas A&M University, College Station, TX, USA
2. Department of Oceanography, Texas A&M University, College Station, TX, USA
3. Key Laboratory of Physical Oceanography and Frontiers Science Center for Deep Ocean Multispheres and Earth System, Ocean University of China, Qingdao, China
4. Qingdao Pilot National Laboratory for Marine Science and Technology, Qingdao, China
5. Department of Atmospheric Sciences, Texas A&M University, College Station, TX, USA
6. College of Global Change and Earth System Science, Beijing Normal University, Beijing, China
7. Department of Geological and Atmospheric Sciences, Iowa State University, Ames, IA, USA
8. These authors contributed equally: Xue Liu, Xiaohui Ma

Correspondence to: maxiaohui@ouc.edu.cn

This PDF file includes:

Supplementary Figs 1-8

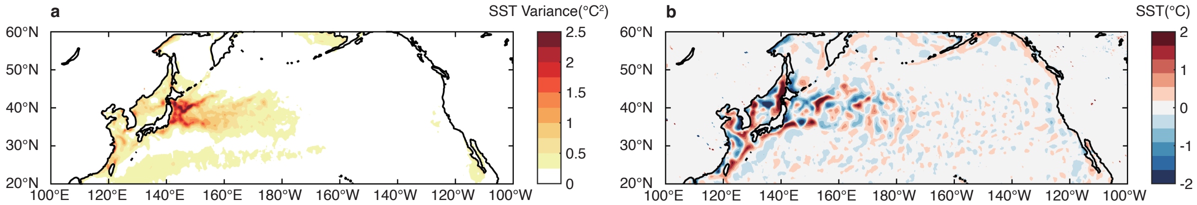

Supplementary Fig. 1 | Mesoscale sea surface temperature (SST) forcing in seasonal-ensemble (SE) and cyclone-ensemble (CE) simulations. Variance of SST difference between SE control (CTRL) and filtered (FILT) for all 13 winter seasons (2002-2014 NDJFM) (**a**). Mean SST difference between CE CTRL and FILT for all 568 cyclone cases (**b**).

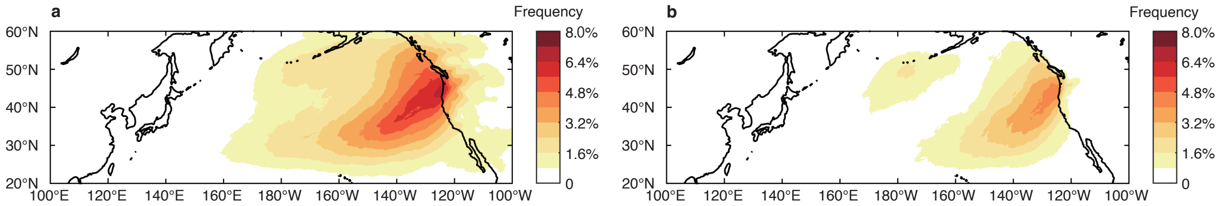

Supplementary Fig. 2 | Simulated and observed landfalling atmospheric rivers (ARs) occurrence frequency. Frequency of landfalling ARs in seasonal-ensemble control (SE CTRL) (**a**) and in reanalysis data (ERA5, 1979-2017) during boreal winter season (NDJFM) (**b**). The frequency is defined as the number of days when landfalling ARs occur divided by the total number of winter season days.

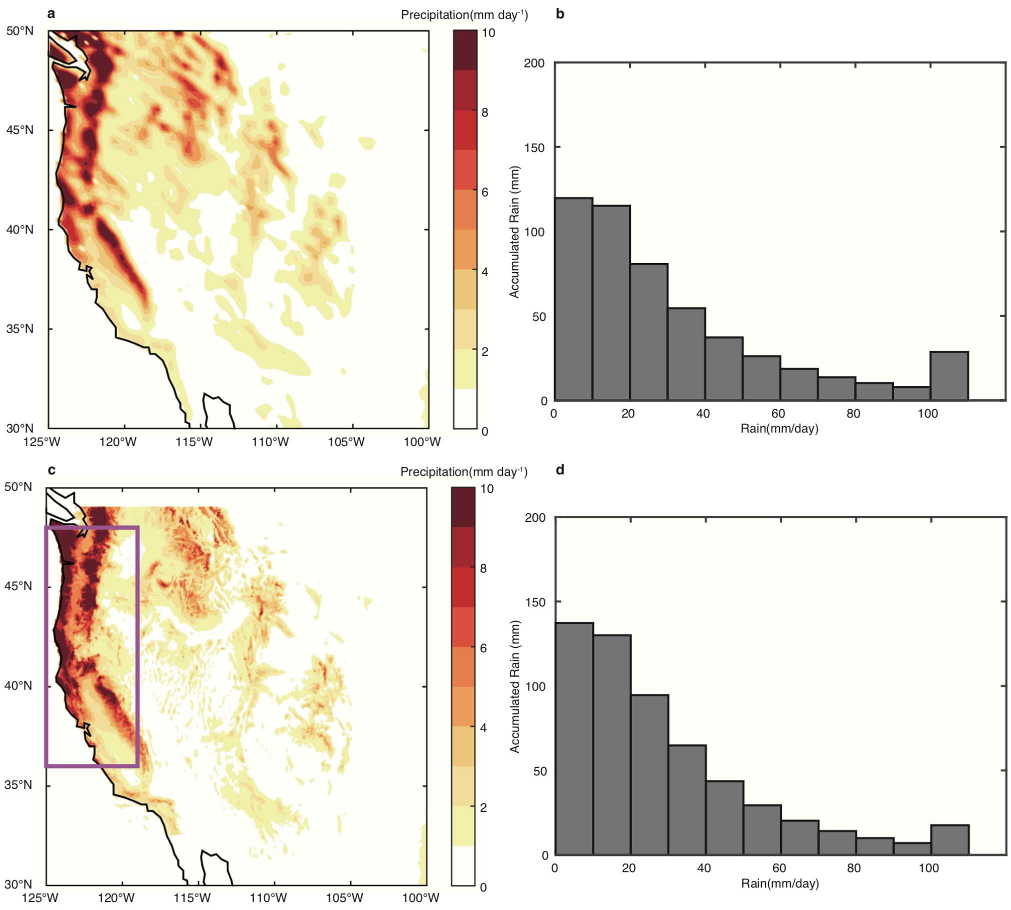

Supplementary Fig. 3 | Simulated and observed winter precipitation over the western United States (US). Winter season mean (NDJFM) precipitation in seasonal-ensemble control (SE CTRL) (**a**) and in observed precipitation data (PRISM,1981-2017) (**c**). Daily precipitation probability density functions (PDFs, averaged in the magenta box in **Supplementary Fig. 3c**) in SE CTRL (**b**) and in PRISM (**d**).

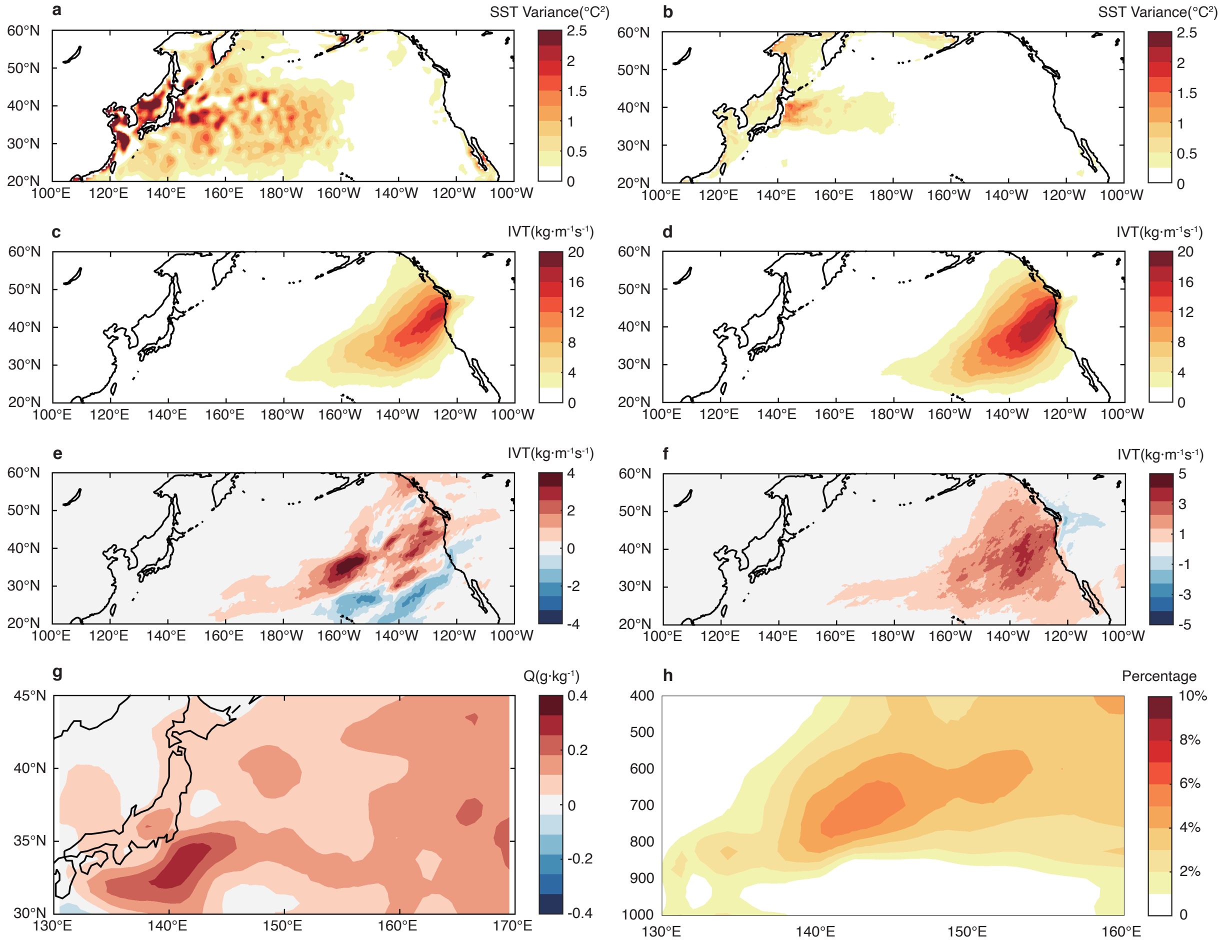

Supplementary Fig. 4 | Response of landfalling atmospheric rivers (ARs) and water vapor to mesoscale sea surface temperature (SST) forcing in two reanalysis data (ERA-Interim and ERA5). Variance of SST difference between the 0.5°-and-finer SST forcing period (2002-2017) and the 1° SST forcing period (1986-2001) in ERA-Interim (**a**). Accumulated integrated water vapor transport (IVT) associated with landfalling ARs averaged in winter season (NDJFM) during the high-resolution SST forcing period (2002-2017) (**c**) and the corresponding difference between high-resolution SST forcing period (2002-2017) and low-resolution SST forcing period (1986-2001) in ERA-Interim (**e**). **b**, **d**, **f**, as for **a**, **c**, **e**, but for the difference between ERA5, where higher resolution (0.25°) SST forcing was used, and ERA-Interim during the period of 1979-2006. Difference of Q at 800hPa (**g**) and fractional difference of Q along a vertical section of the KE (35°N) (**h**) between high-resolution SST forcing period (2002-2017) and low-resolution SST forcing period (1986-2001) in ERA-Interim. Landfalling AR IVT in **c-f** is computed as the sum of IVT associated with landfalling ARs divided by the total number of all winter season days in ERA-Interim and ERA5, respectively.

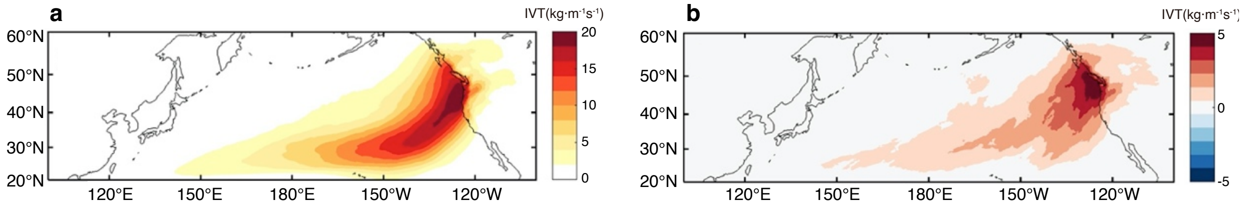

Supplementary Fig. 5 | Response of heavy precipitation associated landfalling atmospheric rivers (ARs) to mesoscale sea surface temperature forcing in the cyclone ensemble experiment. Landfalling AR integrated water vapor transport (IVT) accumulated over the heavy precipitation days averaged over the two-week period in cyclone-ensemble control (CE CTRL) (**a**) and the corresponding difference between CE CTRL and filtered (FILT) (**b**). Landfalling AR IVT is calculated the same as that in **Fig. 2a**.

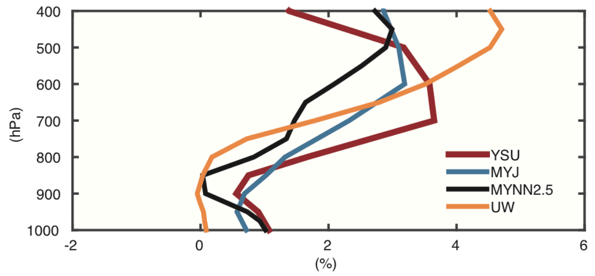

Supplementary Fig. 6 | Sensitivity of water vapor response to different planetary boundary layer (PBL) and surface layer schemes in model (WRF) sensitivity ensemble simulations. Fractional difference of the vertical profile of winter season mean (NDJFM) Q averaged in atmospheric river (AR) genesis region ([25°N-35°N 130°E-150°E]) between control (CTRL) and filtered (FILT) in reference to CTRL of WRF sensitivity experiments with different PBL and surface layer schemes. All the results are based on the ensemble mean of 10 2007/8 winter-season runs in each sensitivity experiment.

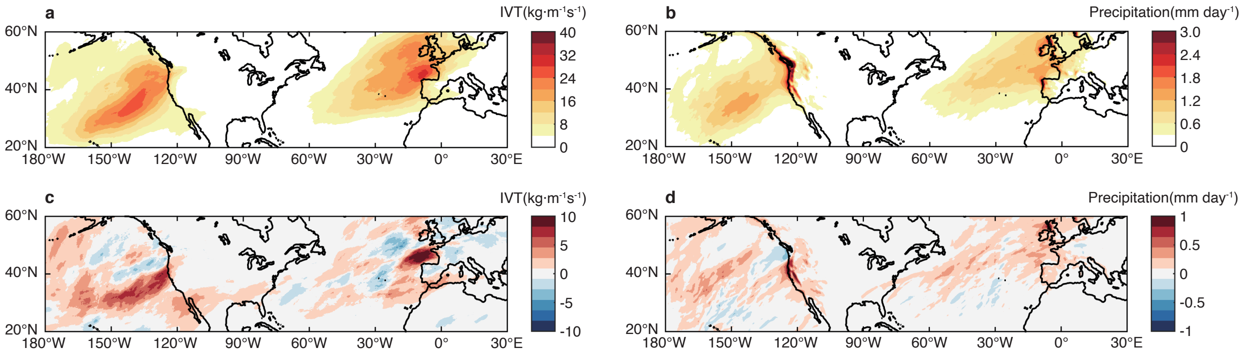

Supplementary Fig. 7 | Response of landfalling atmospheric rivers (ARs) to mesoscale sea surface temperature forcing from global model (CAM) ensemble simulations. Averaged integrated water vapor transport (IVT) associated with landfalling ARs in boreal winter months (DJF) in the control (CTRL) (**a**) and the corresponding difference between the CTRL and filtered (FILT) (**c**). **b** and **d**, same as **a** and **c**, but for precipitation. Averaged IVT (precipitation) is computed as the sum of IVT (precipitation) associated with landfalling ARs divided by the total number of winter month days in global CAM ensemble.

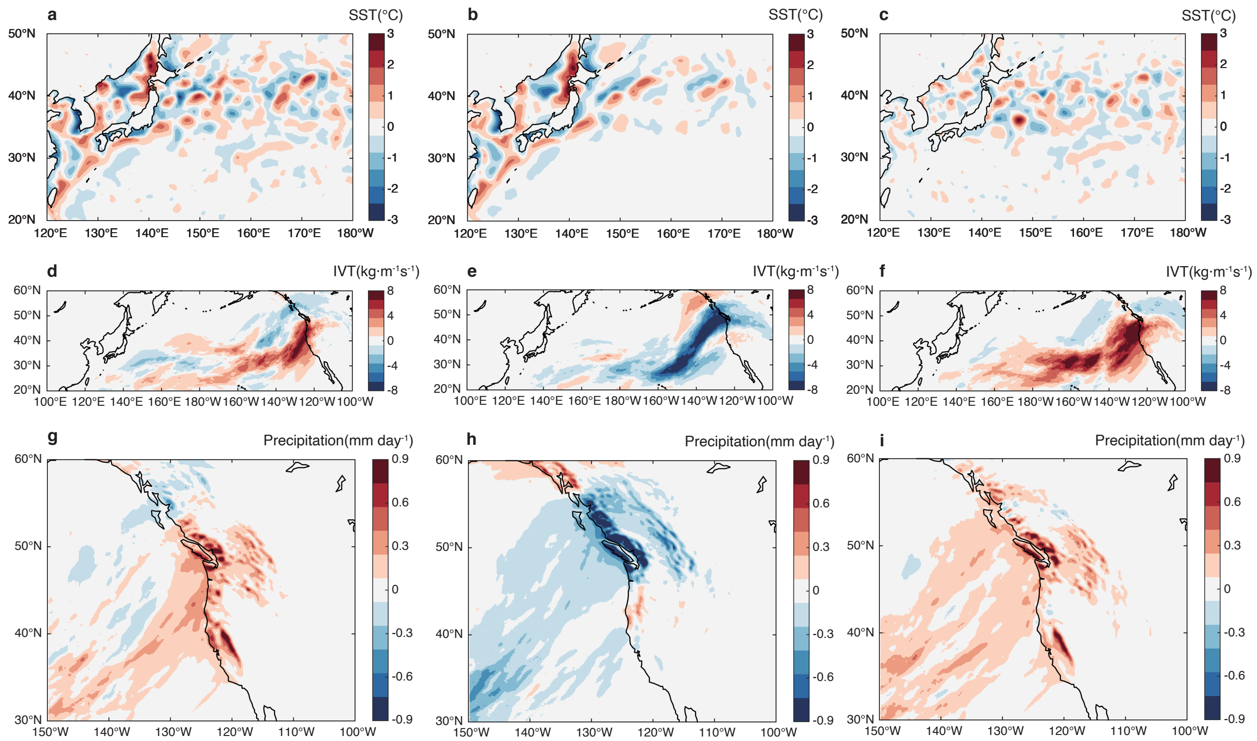

Supplementary Fig. 8 | Relative importance of ocean eddies vs. sea surface temperature (SST) fronts in driving the atmospheric river (AR) response. 2007/8 winter-season mean SST differences in the Kuroshio Extension region between control (CTRL) and filtered (FILT) in front-eddy (F-E) (a), front-only (F-O) (b) and eddy-only (E-O) (c) ensembles. d&g, same as b&d in Fig. 2 in the main text, showing the differences of landfalling AR related integrated water vapor transport (IVT) and heavy precipitation between CTRL and FILT in F-E, respectively. e&h, same as d&g, but for F-O. f&i, same as d&g, but for E-O.
